# Langerhans Cell Histiocytosis Involving the Thymus and Heart With Simultaneous Thymoma: A Case Report

**DOI:** 10.3389/fonc.2022.890308

**Published:** 2022-04-25

**Authors:** Ting Ji, Yuxia Zhong, Deyun Cheng

**Affiliations:** Department of Respiratory and Critical Care Medicine, West China Hospital of Sichuan University, Sichuan, China

**Keywords:** Langerhans cell histiocytosis, thymoma, thymus, heart, ^18^FDG-PET/CT

## Abstract

Langerhans cell histiocytosis (LCH) is a rare disease characterized by clonal expansion of CD1a+/CD207+ cells in lesions. The most frequent sites involved are bone and, less commonly, lymph nodes, lungs, and skin. The thymus or heart is rarely involved with LCH. In this case, we present a 73-year-old woman with a mediastinal mass. Histopathology after thymectomy identified this mass as type AB thymoma; notably, subsequent immunohistochemical tests showed lesions of LCH scattered in the region of thymoma. 18-Fluorodeoxyglucose PET/CT (18-FDG-PET/CT) was performed to make an overall assessment of the extent of this disease, which demonstrated suspicious cardiac involvement of LCH. This report highlights the importance of differentiating abnormalities of the thymus or mediastinal mass from LCH and the necessity of comprehensive evaluation for patients with LCH.

## Introduction

Langerhans cell histiocytosis (LCH) is a rare disease characterized by clonal expansion of CD1a+/CD207+ cells, which often affects children and young adults (1). Thymoma, as the most common neoplasm in the anterior mediastinum, originates from epithelial cells in the thymus regardless of the presence or abundance of lymphoid component (2). It is extremely rare for two lesions of LCH and thymoma to occur in the same organ. Herein, we present a 73-year-old woman diagnosed with thymic LCH concurrent with type AB thymoma after thymectomy, with suspicious cardiac involvement of LCH demonstrated by 18-fluorodeoxyglucose PET/CT (^18^FDG-PET/CT). We believe that LCH should be included in the differential diagnosis of thymic abnormalities or mediastinal masses.

## Case Presentation

**Figure 1:**
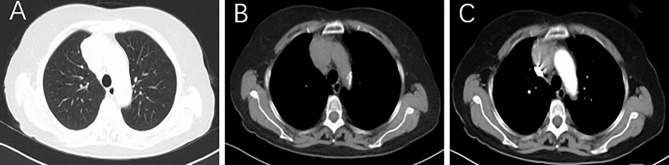
Lung **(A)** and mediastinal window **(B)** of axial CT images show an irregular mass (about 5.1 × 3.5 cm) in the right anterior upper mediastinum, with inhomogeneous enhancement on contrast-enhanced CT **(C)**.

**Figure 2:**
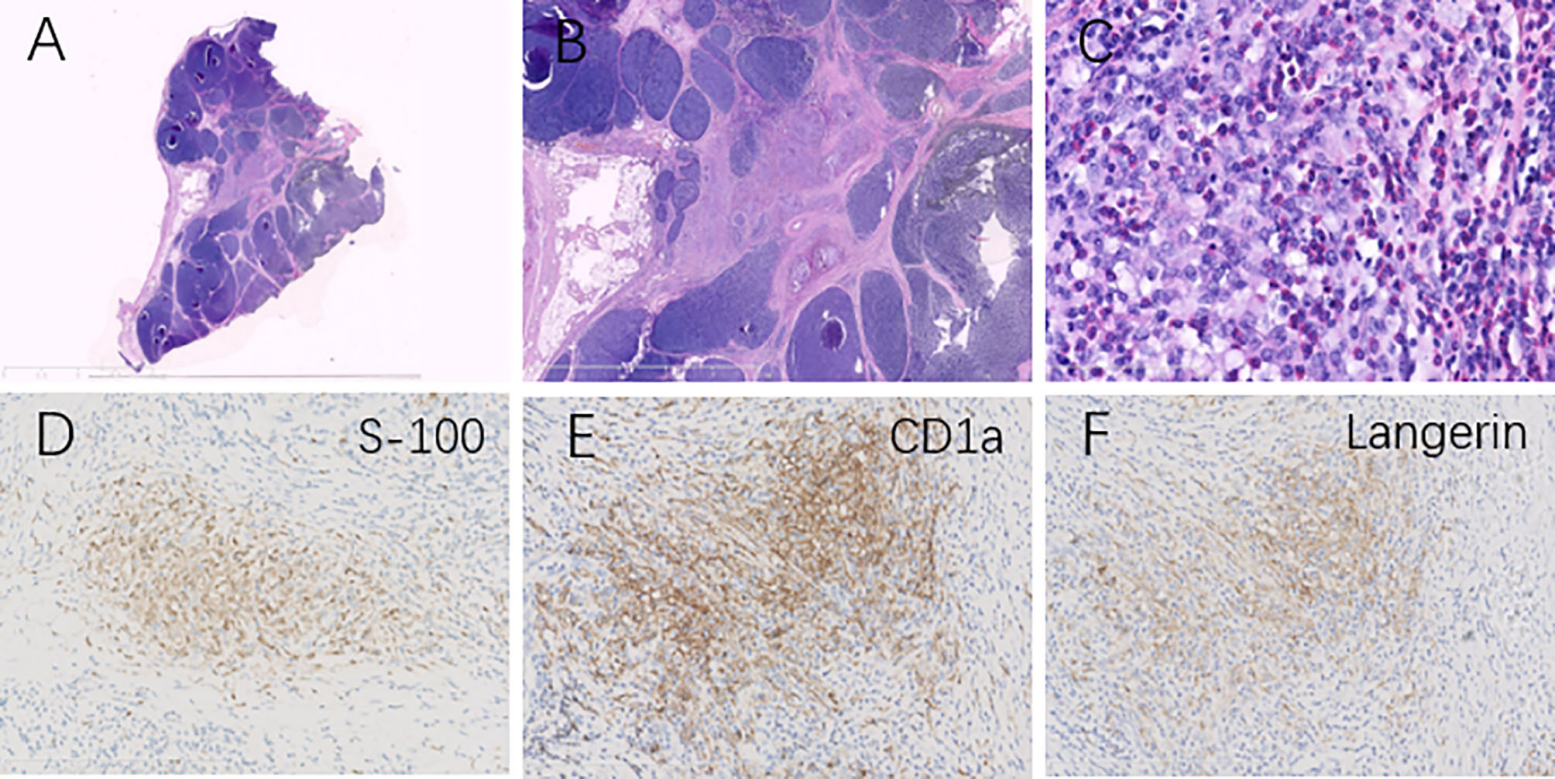
Histopathological analysis revealed type AB thymoma composed of a lymphocyte-poor area and lymphocyte-rich area at low magnification **(A, B)**. Clusters of mononuclear cells and eosinophils **(C)** were surrounded by lesions of thymoma (H&E, magnification ×400), and immunohistochemical tests showed these clusters were positive for S100 **(D)**, CD1a **(E)**, and Langerin **(F)**, identifying the diagnosis of Langerhans cell histiocytosis in the thymus.

**Figure 3:**
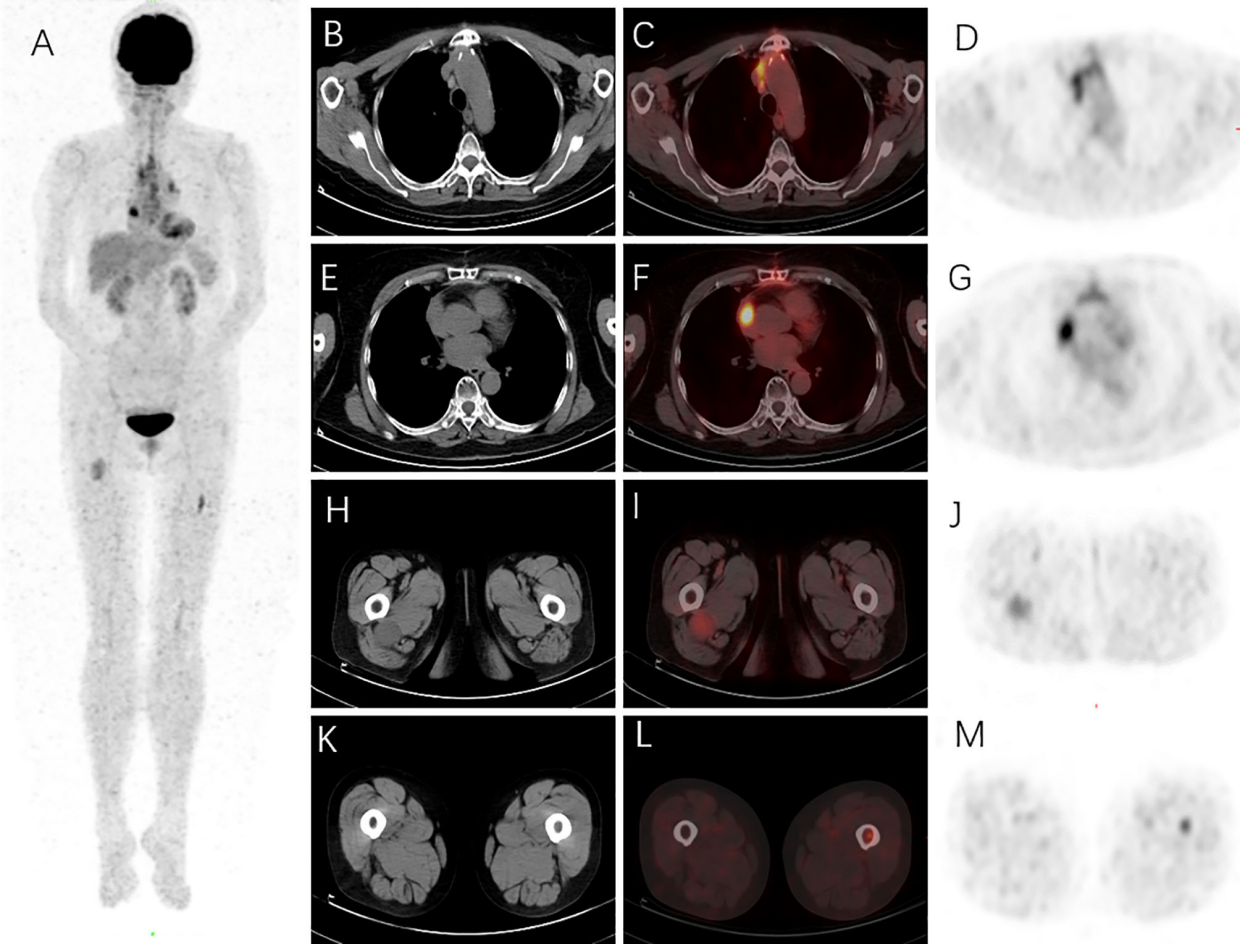
^18^F-FDG PET/CT was performed on this patient to evaluate the extent of the Langerhans cell histiocytosis (LCH). The maximum intensity projection (MIP) revealed increased ^18^F-FDG uptake in the chest, left femur, and right thigh regions **(A)**. Surgery-related changes of the sternum on the mediastinal window of axial CT **(B)** were shown with diffusely high ^18^F-FDG uptake and SUVmax as 4.08 on axial PET/CT fusion **(C)** and PET images **(D)**. An ^18^F-FDG-avid lesion in the right atrial appendage was found with SUVmax as 7.08 on axial PET/CT fusion **(F)** and PET images **(G)**, and no specific abnormal density was found in the corresponding region on the mediastinal window of axial CT image **(E)**. A round mass (about 3.7 × 3.1 cm) in the right upper thigh on the soft tissue window of axial CT **(H)** was revealed with SUVmax as 3.20 on axial PET/CT fusion **(I)** and PET images **(J)**. A hypermetabolic lesion in the left upper femur was found with SUVmax as 3.98 on axial PET/CT fusion **(L)** and PET images **(M)**; bone marrow density was slightly increased **(K)**.

A 73-year-old woman presented with chest tightness without evidence of myasthenia gravis and other complaints, such as fever, weight loss, pain, mass, skin lesion, and cough. She had no previous history of carcinoma or other comorbidities. Physical examination showed no abnormal signs. Her blood tests revealed no apparent abnormalities, including blood cell count, renal and liver function, and tumor markers (NSE, CYFRA21-1, CA15-3, CA19-9, and CA12-5). Chest CT showed an irregular mass (51 mm × 35 mm) in the anterior mediastinum, with inhomogeneous enhancement on contrast-enhanced CT (**Figure 1**). A mediastinal tumor was highly suspected, and a biopsy was considered. After the physician communicated with this patient and her family, the patient had chosen direct thymectomy, not biopsy. Histopathology identified this mass as a type AB thymoma composed of lymphocyte-poor areas and lymphocyte-rich areas at low magnification. Small nodules of mononuclear cells and eosinophils were surrounded by lesions of thymoma, and immunohistochemical tests showed that these nodules were positive for S100, CD1a, and Langerin (**Figure 2**). The genomic analysis revealed no BRAF^V600E^ mutation. Therefore, thymic LCH co-occurrence with type AB thymoma was considered. This patient also received radiotherapy after thymectomy (GTV5500cGy/25f, CTV5000cGy/25f). ^18^F-FDG-PET/CT was performed (**Figure 3**) and showed abnormal hypermetabolic regions in the chest, left femur, and right thigh, with the following maximum standardized uptake value (SUVmax): surgery-related changes of the sternum, 4.08; right atrial appendage, 7.08; a round mass with a size of 37 mm × 31 mm at the right upper thigh, 3.20; and left upper femur, 3.98. The patient underwent a biopsy for the mass of the right upper thigh in another hospital, of which immunohistochemical tests also proved the involvement of LCH. We could not be able to define the nature of lesions in the left upper femur and right atrial appendage because of difficulty in the biopsy. Eighteen months after thymectomy, there were no signs of recurrence on the chest contrast-enhanced CT, and the mass of the right upper thigh remains the same size. She also did not complain of symptoms of heart failure and bony pain.

## Discussion

The annual incidence of LCH is 5−9 cases per million in children older than 15 years and 1 case per million in patients older than 15 years (3). Thymoma is also a rare tumor of the mediastinum with an annual age-adjusted incidence of 0.9–2.3 cases per million (2). Therefore, thymoma and LCH rarely occur in the same organ.

LCH commonly affects the bone (80%), skin (33%), pituitary gland (25%), and lungs (15%) (3). The thymus is rarely involved (4). A retrospective study reported that 1.4% of pediatric LCH cases were found to have thymic involvement (5). However, data about thymic LCH in adults are lacking. Patients with thymic LCH could be asymptomatic; some patients are accidentally diagnosed by imaging examination, and others have undergone thymectomy during cardiothoracic surgery and were identified as thymic LCH by pathology (4, 6, 7). In addition, adults suffering from thymic LCH can also present with myasthenia gravis (4), which did not occur in our case. Previous literature had demonstrated the status of thymic LCH combined with lymphoid hyperplasia but no features of thymoma (8). Researchers previously suggested routine imaging screening of the thymus in patients with LCH, especially in young children (5). To some degree, this abnormality of the thymus promotes early diagnosis in our case. We recommend routine screening of the biomarkers of LCH in thymic samples to investigate the presence of LCH.

Cardiac lesions are exceedingly rare in patients with LCH (9). There are no available data for the prevalence of cardiac involvement of LCH. Four case reports have shown the infiltration of the septum (10) and pericardium (11–13) by LCH. In our case, the hypermetabolic lesions in the right atrial appendage and left proximal femur demonstrated by ^18^F-FDG PET/CT were considered neoplastic changes. Due to the high risk of cardiac biopsy, we did not perform it on this patient. Based on the pathological identification of LCH in two regions for this patient, we highly suspect that the hypermetabolic lesions of the heart and femur were caused by LCH. It is true that we must consider the possibility of mixed LCH and Erdheim-Chester disease (ECD) when a patient with LCH has suspicious cardiac lesions (9). However, pericardial infiltration and effusion, sometimes complicated by cardiac tamponade, and pseudotumor of the right atrioventricular groove are the most common regions of cardiac involvement with ECD (9), which is not in line with our case. Due to the steel wire retention sutures in the chest during thoracotomy, cardiac magnetic resonance to help us define the nature of the cardiac lesion in this case cannot be performed on this patient. In most cases, the diagnosis and evaluation of LCH are challenging and delayed because clinical findings are non-specific and complicated. Late diagnosis and assessment could exacerbate LCH and lead to sequelae, such as disability and malformation associated with pathological fracture and growth retardation caused by the progression of LCH in the pituitary and even death (1). Besides, initial evaluation and diagnosis of LCH depend on full-body screening, and all lesions are FDG-avid (9, 14). Therefore ^18^F-FDG PET/CT could be a very useful tool to make a comprehensive evaluation in patients with LCH, guiding therapy for this disease (14, 15).

Currently, although treatment of LCH is not well established, which depends on lesions’ location and number, therapy strategies have been generally agreed upon (1, 16). Single-system lesion confined to only one single site needs local medication, curettage, or observation, such as skin and bone lesion (17). Multiple lesions in single-system LCH (SS-LCH) or multisystem LCH (MS-LCH) require systematic chemotherapy (1, 16). Along with the discovery of the mutated MAPK pathway in the pathogenesis of LCH, targeted therapy by BRAF or MEK inhibitors has become a novel therapeutic strategy for patients with LCH (18). Timely and proper treatment, which prevents the invasion of disease into risky organs (bone marrow, liver, and spleen), could promote a good prognosis (1, 16).

In conclusion, we report an “incidental” thymic LCH combined with thymoma in an old woman with highly suspicious cardiac involvement of LCH and emphasize the necessity of differentiating abnormalities of the thymus from LCH and comprehensive evaluation for patients with LCH, if possible, by PET/CT.

## Data Availability Statement

The original contributions presented in the study are included in the article/supplementary material. Further inquiries can be directed to the corresponding author.

## Ethics Statement

The studies involving human participants were reviewed and approved by the Ethics Committee of the West China Hospital of Sichuan University. The patients/participants provided their written informed consent to participate in this study. Written informed consent was obtained from the individual(s) for the publication of any potentially identifiable images or data included in this article.

## Author Contributions

All authors have made a significant contribution to this paper. TJ and YZ drafted the manuscript. DC revised the manuscript. All authors contributed to the article and approved the submitted version.

## Conflict of Interest

The authors declare that the research was conducted in the absence of any commercial or financial relationships that could be construed as a potential conflict of interest.

## Publisher’s Note

All claims expressed in this article are solely those of the authors and do not necessarily represent those of their affiliated organizations, or those of the publisher, the editors and the reviewers. Any product that may be evaluated in this article, or claim that may be made by its manufacturer, is not guaranteed or endorsed by the publisher.
